# A Novel Knowledge Distillation-Based Feature Selection for the Classification of ADHD

**DOI:** 10.3390/biom11081093

**Published:** 2021-07-23

**Authors:** Naseer Ahmed Khan, Samer Abdulateef Waheeb, Atif Riaz, Xuequn Shang

**Affiliations:** 1 School of Computer Science and Technology, Changan Campus, Northwestern Polytechnical University, Xi’an 710072, China; samirabdulateef@mail.nwpu.edu.cn (S.A.W.); shang@nwpu.edu.cn (X.S.); 2 Department of Computer Science, University of London, London WC1E 7HU, UK; atif.riaz@city.ac.uk

**Keywords:** ADHD, autoencoder, classification, connectivity, features selection, neural networks, fMRI, rs-fMRI

## Abstract

Attention Deficit Hyperactivity Disorder (ADHD) is a brain disorder with characteristics such as lack of concentration, excessive fidgeting, outbursts of emotions, lack of patience, difficulty in organizing tasks, increased forgetfulness, and interrupting conversation, and it is affecting millions of people worldwide. There is, until now, not a gold standard test using which an ADHD expert can differentiate between an individual with ADHD and a healthy subject, making accurate diagnosis of ADHD a challenging task. We are proposing a Knowledge Distillation-based approach to search for discriminating features between the ADHD and healthy subjects. Learned embeddings from a large neural network, trained on the functional connectivity features, were fed to one hidden layer Autoencoder for reproduction of the embeddings using the same connectivity features. Finally, a forward feature selection algorithm was used to select a combination of most discriminating features between the ADHD and the Healthy Controls. We achieved promising classification results for each of the five individual sites. A combined accuracy of 81% in KKI, 60% Peking, 56% in NYU, 64% NI, and 56% OHSU and individual site wise accuracy of 72% in KKI, 60% Peking, 73% in NYU, 70% NI, and 71% OHSU were obtained using our extracted features. Our results also outperformed state-of-the-art methods in literature which validates the efficacy of our proposed approach.

## 1. Introduction

Brain is considered the most intricate and mysterious organ in the human body with complexity in networks in the spatial as well the temporal domain. Brain’s functional and physical levels consists of five major regions: Frontal, Occipital, Parietal, Subcortical, and Temporal regions [1]. The complexity of the human brain is related to both the increasing age and difficulty level of the computational task. Increasing age of the person and the complicated nature of the task make it difficult for the brain to make decisions [2,3]. The volume of data generated by the human brain is huge and this amount of data in just half a minute is equivalent to the data generated by the Hubble telescope in its entire life [4], which makes analysis based on human brain data a challenging task.

Attention Deficit Hyperactivity Disorder (ADHD) is a brain disorder that is characterized with persistent lack of attention, high impulsiveness, restlessness, and hyperactivity with numerous environmental, neurological, and genetic factors [5,6,7]. The rate of ADHD diagnosis is increasing in children and it affects 8% to 12% of the world’s child population as indicated in the studies in [8,9]. A benchmark for the prevalence of ADHD among children using meta-analysis based on 179 estimates of the prevalence in 175 studies is proposed in [10]. There are both genetic- [11] and neurological-related [12] interpretations of the cause of ADHD, specifically, genes LPHN3 and CDH13 and damage to the frontal lobe, respectively.

ADHD on the subjects is measured using a variety of modalities with each modality has peculiar characteristics. These modalities include DTI (Diffusion Tensor Imaging), EEG (Electroencephalography), fMRI (Functional Magnetic Resonance Imaging), PET (Positron Emission Tomography), and SPECT (Single Photon Emission Computed Tomography). Brain structural alterations were observed in proband, alterations in brain functional connectivity and influence of the drug in the treatment of ADHD using the DTI modality were discussed in the studies in [12,13,14]. Diagnostic psychiatry tests for ADHD were based on four steps using the the 17 meta-studies and a meta-analysis based on randomized control trials on the ADHD were discussed in [15,16], respectively. A triple-blinded studies on the 275 children and adolescents to integrate the biomarkers for the diagnosis of ADHD and a deep learning-based framework for the diagnosis of ADHD were discussed in [17,18]. A convolution neural network and a comparisons of alpha powers between the 25 patients and 22 healthy controls for the diagnosis of ADHD using the EEG modality were discussed in [19,20,21], respectively. Effect of psychostimulants on the 16 youths, with modeling of the brain using the resting state of the brain with the help of Independent Component Analysis (ICA) and A meta-analysis of 55 studies involving 55 children and adults were discussed in [22,23,24], respectively. The inter-connections and intra-connections in the brain functional regions, effect of 40 mg methylphenidate on the 37 individuals, and effect of L-theanine (2.5 mg) and caffeine (2.0 mg) on the patients with ADHD using fMRI modality were studied in [25,26,27], respectively. PET-related studies have also shown promising results on the ADHD subjects and the biology of this disorder [28]. A significant increase in Dopamine Transporter (DAT) binding was observed in [29] conducted on 47 subjects with matched control. In [30], alterations in the cortical thickness were found between the ADHD and the healthy controls. One multimodal study [31] using PET and genetic data on the 20 ADHD and matched healthy controls and another machine learning-based study [32] on 16 ADHD subjects and 22 healthy controls found promising results in the diagnosis of this disorder. SPECT-based studies were conducted in [33] to distinguish sub-types of ADHD. A meta-analysis [34] involving 51 studies on 53 ADHD subjects found 13 promising genes for the diagnosis of ADHD using the SPECT-based image scans on the patients. A study on ADHD and other disorders using the SPECT modality showed alterations in some of the brain regions [35]. A genetic SPECT study [36] found a decrease in DAT on the 20 adolescents. A use of SPECT as to how it is aiding the medical treatment of the ADHD subject is discussed in the study [37].

fMRI studies based on functional connectivity on the ADHD are becoming, of late, very popular due the the noninvasive nature of fMRI and interpretable regions found from the extraction of functional connectivity matrix. In this regard, ADHD diagnosis using personal characteristics (age, IQ, and handedness) [38] showed promising results. Independent Component Analysis (ICA) with the combination of functional connectivity matrix found neural network dysregulation in ADHD [39]. Fusion [40] of non-imaging data with the imaging data also showed promising results in the study. fMRI study also found functional connectivity alterations [41] in the right inferior frontal cortex of the adolescents. A study using Convolution Neural Network (CNN) [42] on the multi-site resting state fMRI data showed promising results in the classification of the ADHD .

In this study, we propose a Deep Learning-based approach for the classification of ADHD subjects and healthy controls. Our approach is inspired from the work in [43], as a similar distillation model is conceptualized in that problem, but the novelty of our work is that we have used this model as a part of the bigger pipeline of feature selection. We used knowledge distillation as a first step to find the indices of the most discriminating features from the final feature index vector and after that to selection of the most discriminating features subset using Sequential Forward Feature Selection (SFFS) Approach. Details of our knowledge distillation process is as follows. First a connectivity matrix between the each pair of region on a subject is computed using a “community matrix” which is described in Section 3.3.2 and the lower triangular unique elements excluding the diagonal are extracted from this matrix to be used as an “Input” in our study. In phase one, we trained a large neural network on all the input corresponding to all the subjects and extracted the trained codes of the hidden layer, the idea was that the large neural network should be able to understand the complex structure that existed between the Input and the output condition of ADHD and healthy controls. In the second phase, we trained a one hidden layer-based autoencoder on the input connectivity features and the extracted trained codes on the subjects from the large neural work. Here, the purpose of autoencoder was to be able to learn the underlying structure between the input connectivity features and the trained codes. After that we extracted the weights from the input to hidden layer matrix from the autoencoder and sorted them in descending order of magnitude. The higher the value of the weight, the most likely that feature index correspond to most discriminating feature vector. In the final phase, as we needed a set of most discriminating features, we used a Sequential Forward Feature Selection (SFFS) approach to select the subset of features that were most discriminant using various classifiers and compared our results using the experiments that we described in the experimental subsection. In the sections to follow, we will proceed as follows. First, we will discuss about the related research. Second, we will discuss about the dataset and our proposed methodology. Third, we will discuss in details about the feature selection for the classifiers. After that we will discuss the and interpret the usefulness of he related features, selected using our approach. Last, we will conclude our study, will discuss its limitations and future improvement to solve these issues.

## 2. Related Research

In this section, we have categorized and explained various studies that were conducted on ADHD using the fMRI modality.

### 2.1. Structural Information Based Approaches

In [44], the authors used morphological information to classify 210 ADHD subjects from the 226 healthy controls. They used isotropic local binary patterns on three orthogonal planes to extract features from the high-resolution MRI scan data on the subjects resulting in 69% accuracy. In the study [45], high-resolution 3-D scans of 55 ADHD subjects and matched healthy controls were acquired using MRI machine. After processing them with the FreeSurfer [46] software, 340 features such as cortical thickness, curvature, volume, etc. were measured for each type of subjects resulting in maximum accuracy of 90.18% when given to Extreme Learning Machine classifier. Gaussian Process Classification was applied in [47] to the brain gray matter volumetric data including 29 ADHD and matched control subjects resulting in overall accuracy of 79.3%. Structural as well as functional features were used in [48,49] to classify the ADHD subjects resulting in 76% accuracy in the multi-class setting and 92.8% accuracy in the binary class setting in the first study and 67% accuracy in the second study. A study [50] on 508 individuals containing ADHD subjects and healthy control using the source based morphometry of the brain scans showed alterations in bilateral CrusI and bilateral insula between the two conditions among subjects.

### 2.2. Functional Connectivity Based Approaches

In [51], decreased functional connectivity was observed in dorsal anterior cingulate cortex and regions of default mode network between the 21 ADHD patients and 21 matched healthy controls. In study [52], 20 medication-naive ADHD children with 20 age- and gender-matched healthy controls were investigated for the alterations in functional connectivity and found delayed maturation in two functional networks. In  [53], functional connectivity alterations in the brain areas related to motor circuitry which contribute to the functioning of motor and attention were exhibited in children. A Fully Connected Cascade (FCC) neural network was proposed in [54] to discriminate ADHD from the healthy controls, and directional and non-directional based connectivity features were given to the classifier resulting in 90% accuracy. In  [55], involving 20 ADHD patients and 27 healthy controls, increased connectivity in the brain Default Model Network (DMN) was found both between and among the functional connectivity networks. In  [56], from the data on 95 ADHD subjects and 90 healthy controls, the authors selected five subcortical regions. Their analysis showed significant difference in resting state functional connectivity in caudate nucleus. In  [57], the authors formed two cohorts: a child cohort consisting of 34 ADHD and 28 health controls and an adult cohort consisting of 112 ADHD and 77 healthy controls. Functional connectivity alterations were found both in the children cohort and in the adult cohort. A multi-objective scheme using Support Vector Machine (SVM) was used in [58] to first tackle the task of imbalanced dataset and then classifying the ADHD subjects from the healthy controls with promising results. The dual subspace method was observed in [59] by first making two subspaces corresponding to ADHD and healthy control and then using them based on the energy principle to classify ADHD from the healthy controls.

### 2.3. Deep Learning-Based Approaches

Deep learning is a computational model using which we learn multilevel abstraction of the input data and learn the intricate pattern from the data by training layer wise feed forward neural network with back-propagation algorithm [60]. Deep learning is closely associated to machine learning which has applications in various practical domains such as IoT, renewable energy, medicine, and agriculture [61,62,63,64]. Of late, deep learning is being used increasingly more in the medical image analysis domain to replace handcrafted features with automatic extracted features [65,66]. In [67], the authors discussed scenarios where the subdomains of deep learning including computer vision, natural language processing and reinforcement learning can be applied in the healthcare setups. A 4D-CNN-based algorithm was proposed in [68] with data augmentation for balancing to extract both spatial and temporal features from the ADHD subjects and healthy controls resulting in 71.3% accuracy. DeepFMRI was proposed in [69], three networks—a feature extractor network, a functional connectivity network, and a classification network—were all combined into one big network to form an End-To-End approach with promising results across three ADHD sites. In  [70], a Convolution Denoising Autoencoder (CDAE) was used to extract the discriminating features between the ADHD subjects and healthy control and then the Adaptive boosting Decision Trees (AdaDt) was used for classification using the extracted Features.

## 3. Materials and Methods

### 3.1. Dataset

The dataset used in this study is ADHD-200 [71], which is compiled by the International NeuroImaging Datasharing Initiative (INDI) consortium, consisting of eight international imaging sites around the world. This dataset consists of 776 training and 197 testing subjects with information on gender, age, IQ, and handedness. The dataset that we used consists of five different sites out of total 8 sites which are Kennedy Krieger Institute (KKI), NEUROIMAGE Sample (NI), New York University Child Study Center (NYU), Peking University (PEK), and Oregon Health and Science University (OHSU). The INDI consortium has also provided the testing dataset for the six sites, for comparison with state-of-the-art methods we have only used dataset from the five sites. Details of training and testing dataset along with phenotypic condition is presented in Table 1.

### 3.2. Preprocessing of the Dataset

The ADHD-200 dataset was preprocessed with the Neuroimaging Analysis Kit (NIAK). The steps of the preprocessing include, slice time correction, motion correction, coregistration, normalization, quality control, spatial smoothing, and many others as described in [72,73,74]. Moreover, each of the sites has different scanning parameters that they used for the participants and varying inclusion, exclusion, and update criteria. For example, OHSU site has problem in four subjects in T1-fMRI coregistration step they made adjustment with alignment and after that updated those four subjects.

### 3.3. Methodology

#### 3.3.1. Affinity Propagation

Clustering is a crucial step in forming the community matrix that we used as the input in our proposed approach. Clustering approaches suffer from the drawback of pre-specifying the number of clusters to be formed out of the dataset. We have used Affinity Propagation [75] clustering message passing approach in our algorithm that does not need pre-specification of number of clusters. An important consideration in using Affinity Propagation is the value of preference as this value affects the number of clusters. In our proposed approach, we set preference value from negative −10,000 to +10,000 in increments of 100 so that the complete distribution of the preference value is taken into consideration when calculating the community connectivity matrix.

#### 3.3.2. Architecture of the Proposed Approach

**Figure 1:**
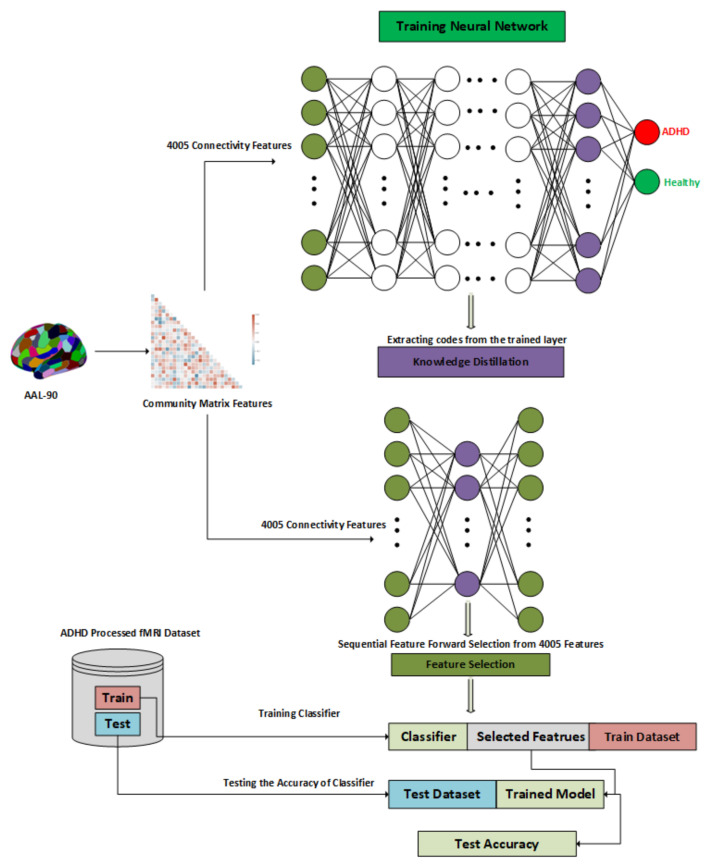
Proposed architecture of the feature selection approach.

The detailed architecture of the proposed approach is displayed in Figure 1. Our proposed approach works by training of a large neural network to learn the representation of the features, an autoencoder for features reconstruction and finally a feature selection method that we explain in the following three sections.

#### 3.3.3. Latent Representation of Dataset

We converted the subject matrix corresponding to the ADHD and Control subjects into the feature vector that were extracted using the community matrix and trained a large neural network based on these features on the whole dataset as displayed under the title of “Training Neural Network” in Figure 1. The core purpose of training a relatively large neural network was to learn the trained embeddings corresponding to the ADHD and Control subjects that could be used in the later stage of knowledge distillation.

#### 3.3.4. Knowledge Distillation

After learning the trained embeddings on the dataset, we used a one-layer Autoencoder with the output layer set to the trained embeddings and the input layer set to the community based features. This is called the knowledge distillation phase as we want our features get reconstructed based on the bottleneck layer so that discriminating features could be sorted as displayed under the title of “Knowledge Distillation” in the Figure 1.

#### 3.3.5. Sequential Forward Feature Selection

Finally, a sequential features selection algorithm, which will select the features in stepwise increments, is used. It selected the most discriminating features corresponding to ADHD and Control subjects as displayed under the title of “Features Selection” in the Figure 1. The reason we want to use the sequential feature selection approach is that we do not want to miss the discriminating features.

### 3.4. Algorithms

We describe now the algorithms that are used in our proposed approach.

#### 3.4.1. Connectivity Matrix

We have used a connectivity matrix based features as the input to our model, that is described in detail in the Algorithm 1. Here, first a clustering of the subject region wise time series is done using the Affinity Propagation [75] which will assign the clusters to each of the 90 regions of the subjects which we can name as a region clustered labels. Then, a matrix of 90 × 90 regions is constructed based on the similarity of the labels of the region clustered labels. The reason of using Affinity Propagation or message passing algorithm is that it does not require predefined number of clusters. In this algorithm, “X” is the input connectivity matrix, “P” is the preference value and “AffProp” is the Affinity Propagation algorithm.
**Algorithm 1** Connectivity Matrix  **Input:** $X_{M\times N}$, *P*  **Output:** $C_{R\times R}$1:$C_{R\times R}\leftarrow 0$2:$Y_{R}\leftarrow AffProp\left(X_{m\times n},P\right)$3:**for**i=0toR**do**4:  **for** j=0 to R **do**5:   **if** $Y_{i}$ = $Y_{j}$ **then**6:   
$C_{i,j}=1$7:   **else**8:   
$C_{i,j}=0$9:   **end if**10:  **end for**11:**end for**

#### 3.4.2. Community Matrix

A correlation matrix based on the Pearson correlation [76] is constructed for the connectivity matrices; however, here we have used the community matrix as described in [77] instead. The details of the community matrix are also described in the Algorithm 2 where different connectivity matrix based on the change in the “preference” parameter in the Affinity propagation is used to construct a number of connectivity matrix which are then averaged together. The core reason for using the community matrix instead of the correlation matrix as described in the study [77] is the sparseness of the community matrix. Moreover, the value of the community matrix corresponding to the two brain regions measures the probability of those two regions to be in the same cluster. Connectivity matrices are usually constructed using the Person’s correlation coefficient and they are easy to calculate. However, two regions which are not connected still have a value corresponding to their correlation which makes it difficult for the classifier to ignore this value. On the other hand, community-based connectivity matrices are calculated using the clustering method. Therefore, two regions which are in the same cluster are more probable to be connected. Due to clustering the community matrix based connectivity matrix is more sparse and make it easy for classifier in learning the pattern of the latent dimension from the data. In this algorithm “X” is the input connectivity matrix and “P” is the preference value of the Affinity Propagation algorithm.
**Algorithm 2** Community Matrix Features  **Input:** $X_{M\times N}$, PArray  **Output:** $M_{R\times R}$1:$M_{R\times R}\leftarrow 0$2:**for**i=0toLen(PArray)**do**3: 
$P\leftarrow PArray_{i}$4: 
$C\leftarrow ConnectivityMatrix(X_{m\times n},P)$5: 
M←M+C6:**end for**7:M←M/Len(PArray)8:M←LOWERTRIA(M)

#### 3.4.3. Ranking of Features

The details of features ranking are explained in Algorithm 3, where the first community matrix features are fed to a large neural network and the learned codes extracted from the large neural network are fed to a one layer Autoencoder, and finally a ranking of the features is obtained using a simple Argsort method. In this algorithm ‘X’ is input connectivity matrix, ‘PArray’ is the list of preference values, and ‘LOWERTRIA’ is the lower triangular elements of the square matrix.
**Algorithm 3** Features Ranking Algorithm  **Input:** $F_{M\times N}$  **Output:** $I_{N\times 1}$1:$CODES_{M\times N}\leftarrow TrainingNeuralNetwork\left(F_{M\times N}\right)$2:$W_{M\times D}\leftarrow KnowledgeDistill\left(F_{M\times N},CODES_{M\times N}\right)$3:$SCORES\leftarrow Diag(W.W^{T})$4:I←ARGSORT(SCORES)

#### 3.4.4. Features Subset Selection

ADHD is a disease in which not just one region but a subset of regions are involved, as described in the “Introduction” section. Therefore, after ranking the features we need to find a subset of the most discriminating features between ADHD and Controls which is explained in Algorithm 4, where a stepwise forward feature ranking algorithm is used to extract the most discriminating subset of features which could apart Healthy controls from the ADHD. In this Algorithm, ‘X’ is input connectivity matrix, ‘I’ is the indexed of the features, CLF’ is a classifier, and ‘ACCURACY’ is 10-fold accuracy.
**Algorithm 4** Sequential Feature Selection  **Input:** $X_{M\times N}$, $I_{N\times 1}$,CLF  **Output: *F***1:featuresBox←Empty2:accList←Empty3:**for**i=0   to Len(I) **do**4:  features←I[0toi]5:  DataMatrix←X[features]6:  ACC←ACCURACY(CLF[DataMatrix])featuresBox.append(features)accList.append(ACC)7:**end for**8:maxIndex←ArgMax(accList)9:F←featurexBox[maxIndex]

### 3.5. Experimental Settings

A challenging area in training a Neural Network is the setting the values of various hyperparameters of the model. In this study, we used two types of neural network for training and Knowledge Distillation. For learning a latent representation of all the data we used a 13 layer Neural Network model with the input layer set to 4005 connectivity features units and the output layer set to 1 ‘sigmoid’ units corresponding to the ADHD disease presence or absence. The hidden layers of this model were set to 3000, 2500, 2000, 1500, 1000, 500, 100, 50, 10, 5, and 2 units, respectively. A dropout of 0.3 in the initial 3 hidden layers and then 0.2 and 0.1 were used in the later layers. We used ‘tanh’ activation function so that negative values are not clipped away, a batch size of 16, an epoch size of 1000, and optimizer ‘adadelta’ with a learning rate of 0.01 was used for training this large neural network. The combinations of these hyperparameters were used after observing too many oscillations in loss and slow convergence, which are typical in training neural networks. After training, these neural networks codes were extracted from layer 10 of this network and then an Autoencoder with 4005 connectivity units, a hidden layer of 100 units, and the output layer of the trained 10 codes were used for the training with the same hyperparameters settings as in the Neural Network with the exception of optimizers which was set to ‘mse’ as this time task was not classifications but of reconstruction. Note that extraction of codes from a specific layer is a challenging task, as layers with too many parameters or with too few parameters do not produce good reconstruction results. Another justification for selecting the layer from the later layers of the trained neural network is that the final layers of the neural network capture a more abstract representation of the input data in the latent dimension.

## 4. Features Selection

**Figure 2:**
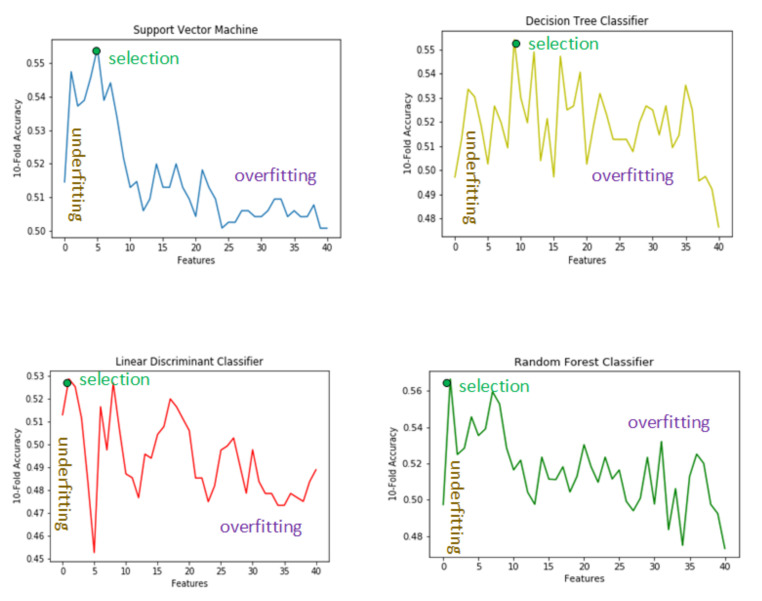
Feature selection approach.

The proposed feature selection philosophy using various classifiers is explained in the Figure 2 where the x-axis represents the number of features(4005) and the y-axis represents the 10-fold accuracy. We have employed various classifiers to check the validity of our feature selection approach so that the robustness of our feature selection approach be emphasized. It can be observed using Figure 2. Initially, the accuracy is low because of the lesser number of features then the accuracy increases for the classifiers until it reached the maximum level and from there it again starts to decrease. In the following three sections we will explain the reasons for having such a phenomena in the case for all the classifiers.

### 4.1. Low Number of Features

A low features set represents the case of underfitting which is a common problem in all those statistical models where the complex phenomena is to be modeled on the given data containing large number of features. In the under-fitting scenario the model is too simple to represent the complex relationship that exists in the problem under hand. As can be seen in Table 2, as the number of features or features set size increases, so does the accuracy of the classifiers; this validates the phenomena of underfitting or bias of the machine learning models. This increasing accuracy corresponding to features set size in all the classifiers thereby ascertaining our assertion of the presence in bias in the model.

### 4.2. Large Number of Features

Similarly, having a higher number of features set represents the typical overfitting scenario, that is, when the model becomes too complex that it cannot correctly predict the result for unseen data. It can be seen from Table 3 that there exists an inverse relation between the number of features and the accuracy of the classifier. Therefore, for all the classifiers, as the set of top features set increases, the accuracy of the classifiers decreases. This phenomena validates our assertion of overfitting or high variance that becomes prevalent in case of selection of high number of features.

### 4.3. Selected Features

After adjusting the cases of underfitting and overfitting as described in the previous two subsections we selected the features that resulted in the highest accuracy for the classifiers. The selected features corresponding to the classifiers are tabulated in Table 4.

### 4.4. Support Vector Machine Features

**Figure 3:**
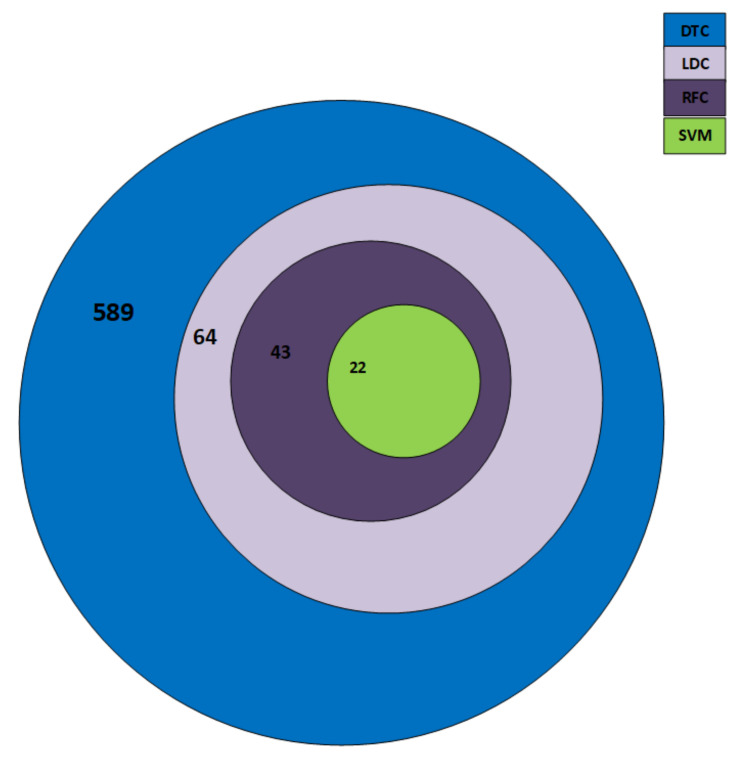
Intersection of features corresponding to classifiers.

As can be observed in Figure 3, those features corresponding to the SVM classifiers are the smallest and those corresponding to the decision tree classifiers are the largest.

Our dataset, as described in Table 1, is imbalanced, that is, there exists unequal cases of ADHD class as compared to HC subjects. SVM has been extensively studied in the literature under the scenario of imbalanced data and there exist optimized decision threshold [78] methods and kernel scaling [79] techniques that can make SVM perform well in the imbalanced cases. Therefore, based on these observations we selected SVM features, as these features are common in all the classifiers and they validate the robustness of this small number of features. Henceforth, in the following sections we will compare results based on the selected SVM features and discuss the significance of these selected features from the anatomical point of view also in the later section.

## 5. Results

Our proposed feature selection approach has produced promising results when compared with the state-of-the-art methods available in the literature. In the following two experiments, we will describe our results based on the two scenarios that are used in literature for the ADHD classification problem.

### 5.1. Training Using Combining Sites

In the first experiment, we trained our model using all the training dataset available in the ADHD-200. The training dataset corresponding to all five sites in Table 1 was combined, and then for testing, the trained model the testing dataset corresponding to each site in Table 1 was used. It can be seen in the Table 5 that our model performed well on all the sites and it was able to perform well when compared to the state-of-the-art methods. Moreover, the accuracy on the remaining two sites for which the comparison accuracy was not available was also very promising.

### 5.2. Testing on Benchmark Datasets

In the second experiment, we trained the model on the training dataset for each site presented in Table 1 and tested the accuracy of our model on the benchmark testing dataset that is presented in the Table 1. Our accuracy results are promising in all the sites as can been seen in Table 6 and robustness of our features can be validated based on the observations that for other model where the accuracy was highest in one site and lower in other, our model still consistently performed better in all scenarios. The accuracy of SC-CNN-ATT [42] is high in the site NYU but low in the sites NI and Peking. Similarly for the work in [80], the accuracy is very high in the site NYU but low in the other two sites. A repeatable observation can be seen in all other methods presented in Table 6.

## 6. Discussion

**Figure 4:**
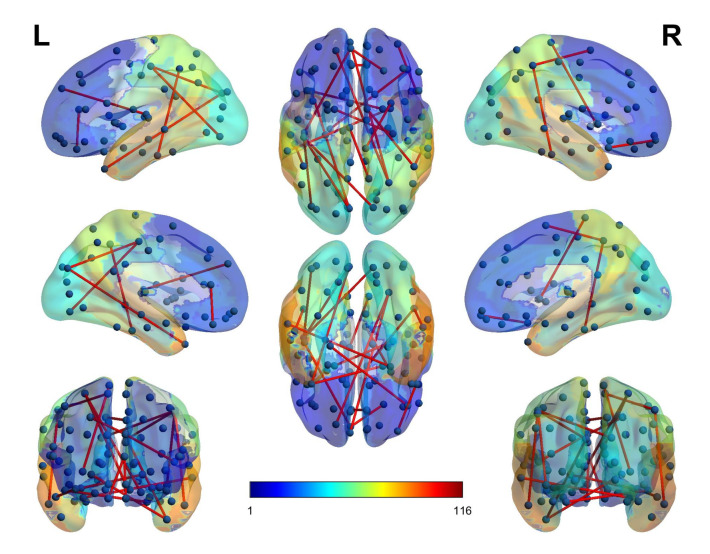
BrainNet visualizations of hemispherical connectivity.

Graph theory [83] is often used in the analysis of various brain disorders using connectivity features. BrainNetViewer [84] is a tool that is employed to visualize the connectivity between the hemispheres as well as the intra-hemispheric regions of the brain. For the BrainNet tool, we need nodes and edges so that they can be visualized. As we used the brain AAL 90 regions atlas, we know out of 4005 features or connectivity regions which pair of regions are connected based on the selected features. Next, the regions that are connected are mapped to the nodes and their existence in the 4005 look-up corresponds to the edge between those two edges. There exists an altered connectivity pattern between the inter-hemispherical and intra-hemispherical connectivity in the two hemispherical regions of the ADHD disorder as can be seen in Figure 4. It can be seen that there is an uneven connectivity pattern in the inter-hemispherical and intra-hemispherical connectivity patterns. Our results are in agreement with the altered connectivity patterns found in [85,86,87,88].

**Figure 5:**
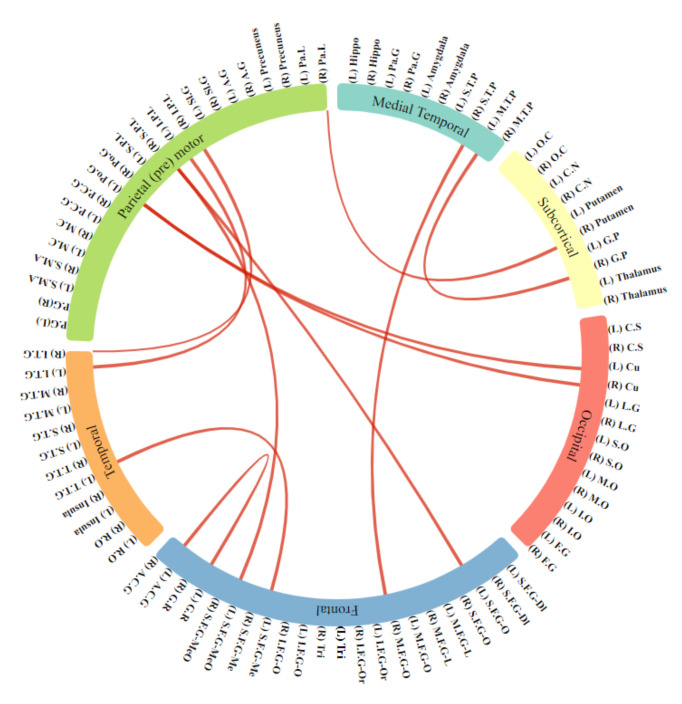
Connectogram for the six regions of the brain.

After observing the connectivity patterns between the hemispherical regions of the brain, we also plotted the connectivity pattern between the six important regions in Figure 5. It can be seen from this connectogram that there are connectivity pattern variations in the brain regions. Moreover, the interconnectivity between the brain regions is also altered in ADHD, which conforms to the previous studies in [40,59,69,89,90].

## 7. Conclusions

We have proposed a feature selection approach in this study on the preprocessed fMRI Dataset on the ADHD brain disorder. Our approach has generated the features that produced the most discriminating and interpretable features when compared with the state-of-the-art methods available in the literature. We have reinforced the idea of functional connectivity in this study, and as per our extensive literature review, this is the first such study where functional connectivity was computed with the help of a community matrix approach and then used in our feature selection pipeline. Our functional connectivity matrix is more sparse and contains less noise that we then used in the knowledge distillation pipeline with the sequential feature selection approach.

### 7.1. Applications

The feature selection technique derived from our knowledge distillation is pretty fast and implementable in all scenarios ranging from slow machines to a comparatively fast machines. There is no bottleneck of high-performance system requirements for our methodology to work, as in the case of other deep learning-based approach. As the dataset corresponding to each site is not big enough for practical purposes, we suggest that our technique should be validated on the large dataset on ADHD so that the robustness and usefulness of the knowledge distillation-based approach could be validated and suggested in practical scenarios.

### 7.2. Future Directions

Of late, End-to-End pipelines are getting more and more attention from the research community in the deep learning paradigm. Therefore, keeping this in view, we believe that an End-To-End deep learning model on ADHD disease based on our proposed approach is an interesting direction to consider. It is no doubt challenging to combine such a heterogeneous pipeline to a one unified model, but we believe that it will open up more opportunities for researchers in knowledge distillation- based approaches working in other domains.

### 7.3. Limitations

We have achieved good accuracy based on our feature selection approaches and our results outperform the best methods available in literature, but still there are two key areas that we need to consider and made progress before this technique could be adapted in studies. First, we believe that the size of the ADHD-200 Dataset is a big issue in training such classification models on ADHD disease. Particularly the availability of data on the sites KKI and NI is far too sparse when compared with the NYU site. Second, the ADHD-200 Dataset is also imbalanced and this can be seen from the data available on the sites KKI and Peking. Based on the above two key issues on the dataset, we are well aware that machine learning models are not well suited in those two cases and lead to biased results in favor of the high number sites which distorts the applicability of the machine leaning models in cases where the Dataset is low in examples.

## Figures and Tables

**Table 1:** ADHD-200 Preprocessed Dataset.

		Training			Testing		
**Sr**	**Imaging Site**	**ADHD**	**HC**	**Total**	**ADHD**	**HC**	**Total**
1	KKI	18	51	69	3	8	11
2	NI	25	23	48	11	14	25
3	NYU	118	98	216	29	12	41
4	OHSU	37	42	79	6	28	34
5	Peking_1	24	61	85	24	27	51
	**Total**	**497**				**162**	

**Table 2:** Accuracy comparison of low number of features.

Top Features	SVM	LDC	DTC	DTC
1	0.49	0.5	0.49	0.49
5	0.53	0.55	0.51	0.53
10	0.54	0.56	0.52	0.54
15	0.55	0.56	0.53	0.56
20	0.55	0.57	0.54	0.56

**Table 3:** Accuracy Comparison of High Number of Features.

Top Features	SVM	LDC	DTC	DTC
100	0.55	0.53	0.54	0.53
500	0.55	0.52	0.55	0.53
1000	0.54	0.52	0.52	0.52
1500	0.52	0.51	0.48	0.45
3000	0.3	0.2	0.4	0.3

**Table 4:** Selection of the Features.

Classifier	Selected Features
SVM	22
LDC	43
DTC	589
RFC	64

**Table 5:** Accuracy(%) comparison of trained model using combined and individual sites.

		Trained on Each Site		Trained on Combined Sites	
**Sr**	**Site**	**Proposed**	**DeepFMRI (2020) [69]**	**Proposed**	**DeepFMRI (2020) [69]**
1	NYU	**73**	73.1	56	65.8
2	NI	**70**	67.9	**64**	60
3	Peking	60	62.7	**60**	43.1
4	OHSU	**71**	-	56	-
5	KKI	**72**	-	**81**	-

**Table 6:** Comparison of proposed method with state-of-the-art techniques.

Method	NYU	NI	Peking	OHSU	KKI
**Proposed**	**73.3**	**70**	**73.3**	**71**	**60**
DeepFMRI (2020) [69]	73.1	67.9	62.7	-	-
SC-CNN-ATT (2020) [42]	77.7	75.3	60.4	-	65.2
FCNet (2018) [40]	67.4	72.9	25.4	-	85.3
Nunez et al. (2015) [81]	-	-	56	-	58
AJHao et al. (2015) [82]	64.7	-	66.3	-	59
Dey et al. (2014) [80]	81	-	56	-	58
ADHD-200 (2012) [38]	-	56.9	35.1	-	51

## Data Availability

ADHD-200 Dataset is publicly available for download at http://preprocessed-connectomes-project.org/adhd200/.

## Abbreviations

The following abbreviations are used in this manuscript:

ADHD	Attention Deficit Hyperactivity Disorder
DTC	Decision Tree Classifier
LDC	Linear Discriminant Classifier
RFC	Random Forest Classifier
SVM	Support Vector Machine
rs-fMRI	Resting State Functional Magnetic Resonance Imaging
